# Does G‐ALPS measure glymphatic function? Revisiting construct validity in diffusion‐based glymphatic imaging

**DOI:** 10.1002/alz.71830

**Published:** 2026-09-07

**Authors:** Einstein Francisco Camargos, Raphael Lopes Olegário, Otávio Toledo Nóbrega

**Affiliations:** ^1^ Medical Sciences Graduate Program University of Brasília Brasília Federal District Brazil; ^2^ Center for Older Adult Medicine, Brasilia University Hospital University of Brasília Brasília Federal District Brazil

1

To the Editor,

We read with great interest the recent article by Jamali et al.^1^ who introduced the G‐ALPS index, a genetic programming‐based refinement of the diffusion tensor image analysis along the perivascular space (DTI‐ALPS) index for evaluating cognitive decline and aging in Alzheimer's disease (AD). Their work represents an elegant application of stochastic optimization to neuroimaging and addresses one of the major challenges in glymphatic research: developing a non‐invasive imaging biomarker of glymphatic function in humans.

Historically, the characterization of the brain extracellular space has relied almost exclusively on invasive experimental techniques. Real‐time iontophoresis (RTI), pioneered by Nicholson and Phillips, enabled quantitative measurements of extracellular volume fraction and tortuosity in animal models.^2^ Using this methodological framework, Iliff and colleagues subsequently described a brain‐wide paravascular pathway through which cerebrospinal fluid (CSF) enters the brain along periarterial spaces, exchanges with interstitial fluid (ISF), and ultimately drains through perivenous routes, introducing the concept of the glymphatic system.^3^ While these discoveries transformed our understanding of brain waste clearance, translating these observations into reliable, non‐invasive biomarkers for humans remains an important unresolved challenge.

Against this background, the introduction of G‐ALPS is both timely and innovative. Nevertheless, we believe that the development of increasingly sophisticated computational models should be accompanied by an equally rigorous discussion of construct validity. Genetic programming can optimize mathematical relationships between diffusion‐derived variables and clinical outcomes, but it cannot, by itself, establish that the resulting index truly measures glymphatic function. In the present study, the G‐ALPS formula was derived to maximize its association with cognitive measures, including the Mini‐Mental State Examination (MMSE) and Clinical Dementia Rating (CDR). This optimization demonstrates predictive performance but does not necessarily validate the biological construct that the index is presumed to represent.

Indeed, both the conventional ALPS index and its optimized version may capture biological processes that coexist with, but are not specific to, glymphatic dysfunction. Diffusion measurements around the perivascular space may also reflect age‐related white matter degeneration, cerebral small vessel disease, neuroinflammation, cortical atrophy, or alterations in tissue microstructure. Consequently, there is a potential risk of circular reasoning: if ALPS‐derived metrics are assumed to represent glymphatic function, their association with AD may be interpreted as evidence of glymphatic impairment, although the physiological specificity of the biomarker has not yet been definitively established in humans.

A related issue concerns internal validity. Glymphatic physiology is highly dynamic and is influenced by several systemic conditions beyond age and sex. Experimental and clinical evidence indicates that hypertension reduces arterial pulsatility, one of the principal driving forces of CSF influx, thereby impairing glymphatic transport.^4^ Likewise, diabetes mellitus has been associated with blood–brain barrier disruption, chronic neuroinflammation, impaired aquaprin 4 (AQP4) polarization, and delayed clearance of neurotoxic proteins, including amyloid‐β.^5^ Although the authors appropriately adjusted for demographic variables, the absence of these important physiological covariates leaves the possibility of residual confounding.

Sleep represents another important consideration. Glymphatic transport reaches its highest efficiency during slow‐wave (N3) sleep, when the cortical interstitial space expands substantially, facilitating CSF–ISF exchange.^6^ In this context, reliance on the Pittsburgh Sleep Quality Index (PSQI) deserves careful interpretation. The PSQI is a retrospective, 30‐day subjective questionnaire that may not adequately reflect the acute sleep architecture most relevant to glymphatic activity. Moreover, it has limited sensitivity for identifying disorders such as obstructive sleep apnea, an increasingly recognized contributor to impaired glymphatic clearance and neurodegeneration.

Finally, the cross‐sectional nature of the study necessarily limits causal inference. Although G‐ALPS demonstrated stronger associations with current cognitive status than the conventional ALPS index, its prognostic value remains unknown. Whether reduced G‐ALPS precedes cognitive decline, represents a downstream consequence of neurodegeneration, or merely reflects normal brain aging can only be determined through longitudinal studies. This distinction is particularly important if ALPS‐derived indices are eventually proposed as surrogate endpoints for therapeutic trials, since improvements in an imaging biomarker do not necessarily translate into meaningful clinical benefit.

We congratulate Jamali et al. for providing an innovative computational framework that advances diffusion‐based neuroimaging. As glymphatic imaging continues to evolve, however, improving algorithmic performance should proceed in parallel with rigorous validation of the biological construct being measured.^7^ Only by integrating computational innovation with physiological validation and careful control of relevant confounding factors will diffusion‐derived biomarkers evolve from statistically optimized indices into reliable measures of human glymphatic function.

## Supporting information




Supporting information
